# Monkeypox Virus Partial-Genome Amplicon Sequencing for Improvement of Genomic Surveillance during Mpox Outbreaks

**DOI:** 10.3201/eid3111.250548

**Published:** 2025-11

**Authors:** Jiusheng Deng, Daisy McGrath, Kimberly Wilkins, Luis A. Haddock, Whitni Davidson, Demi B. Rabeneck, Joseph Madden, Vaughn Wicker, Adrienne Amuri-Aziza, Tony Wawina-Bokalanga, Placide Mbala-Kingebeni, Christina L. Hutson, Yu Li, Crystal Gigante

**Affiliations:** Chenega Enterprise Systems and Solutions, LLC, Chesapeake, Virginia, USA (J. Deng); Centers for Disease Control and Prevention, Atlanta, Georgia, USA (J. Deng, D. McGrath, K. Wilkins, L.A. Haddock, W. Davidson, D.B. Rabeneck, J. Madden, V. Wicker, C.L. Hutson, Y. Li, C. Gigante); University of Kinshasa, Kinshasa, Democratic Republic of the Congo (A. Amuri-Aziza, T. Wawina-Bokalanga, P. Mbala-Kingebeni)

**Keywords:** monkeypox virus, mpox, viruses, antimicrobial resistance, amplicon, genome sequencing, coverage, single-nucleotide polymorphisms, surveillance, genomics, zoonoses, Democratic Republic of the Congo

## Abstract

Mpox is a reemerging infectious disease caused by monkeypox virus (MPXV). Whole-genome sequencing provides comprehensive surveillance of MPXV but is challenging in resource-limited outbreak settings and on clinical samples with low viral load. We developed a low-cost, high-throughput partial-genome sequencing strategy and a freeware Nextflow pipeline for MPXV genomic surveillance. We targeted 2 genomic regions of MPXV by using short overlapping amplicons. This amplicon-based approach generated high-quality sequences over the 2 genomic regions from clinical specimens, including samples with low viral DNA and from formalin-fixed tissues. This partial-genome sequencing approach can determine MPXV subclades and offers an attractive strategy to lower cost and improve MPXV surveillance during outbreaks in mpox-endemic and -nonendemic countries.

Mpox is a reemerging infectious disease caused by monkeypox virus (MPXV) (*1*), a member of the genus *Orthopoxvirus* (*2*). MPXV has 2 distinct clades, clade I and clade II; each clade is further divided into 2 subclades (Ia and Ib, IIa and IIb) on the basis of genetic differences (*3*,*4*). Subclades can be further divided into multiple lineages (https://nextstrain.org/mpox/all-clades), such as lineages A.1, A.2, A.3, and B.1 inside clade IIb (*4*,*5*). 

Mpox has become a new global challenge since the 2022 clade IIb outbreak that led to >100,000 cases across 115 nonendemic countries (*6*,*7*). In addition, the Democratic Republic of the Congo (DRC) and other countries in Africa have reported multiple escalating outbreaks of clade I MPXV over the past several years, including the first clade Ib mpox outbreak (*8*,*9*). The newly identified MPXV subclade Ib has spread within DRC and to neighboring countries (*10*,*11*), and travel-associated exportation has resulted in cases in multiple countries, including in Europe, Asia, and the Americas, through human-to-human close-contact transmission (*12*). Continued cases of clade IIb mpox outside of Africa and ongoing escalation of clade I mpox cases highlight the urgency for the international health community to monitor the disease and to strengthen surveillance of MPXV (*7*,*13*,*14*).

Advanced molecular techniques such as quantitative PCR (qPCR) and whole-genome sequencing (WGS) provide effective approaches for the surveillance of MPXV (*15*,*16*). The MPXV genome has a central conserved core region and 2 variable terminal regions with inverted terminal repeats at both ends (*17*). The 2 variable terminal ends contain clade- and subclade-specific genes, multicopy genes, and low-complexity repeat sequences (*18*), whereas the central core region of each clade has highly conserved genomic sequences encoding essential genes. Orthopoxvirus or MPXV generic qPCRs that target the conserved core region of the genome can rapidly and precisely detect MPXV DNA in samples (*19*); however, those methods might be unable to differentiate clades or subclades of MPXV (*20*). The less-conserved genomic regions that might be targeted by clade- or subclade-specific PCR are more prone to generic drift and deletion mutations that have occurred in poxviruses (*21*). 

WGS has been broadly used to generate consensus genomes and analyze MPXV genomic variation (*6*,*22*), becoming a critical tool for comprehensive surveillance to track circulating and emerging variants, drug resistance, and molecular evolution and to understand the transmission of MPXV (*7*,*23*). However, WGS requires expensive instruments and reagents, as well as samples with high viral load, limiting its use in resource-limited outbreak settings. The ≈200-kb double-stranded DNA genome of MPXV (*24*) introduces challenges for designing and optimizing efficient tiled primers for WGS of all 4 subclades. The conserved core region provides an optimal target for a pool of specific primers for partial-genome sequencing (PGS) of all subclades of MPXV.

In this study, we developed an amplicon-based PGS strategy by targeting 2 genomic regions in the central conserved core region of the MPXV genome (a 10-kb region and a 15-kb region) by using a portable MiniON sequencing device with low-cost Flongle flow cells from Oxford Nanopore Technologies (ONT) (https://nanoporetech.com). We evaluated the specificity of the PGS data to determine MPXV subclades in clinical specimens and the ability to sequence poor-quality specimens to improve the sensitivity of MPXV genomic surveillance.

## Materials and Methods

### Mpox Clinical Specimens

Mpox specimens used in this study included remainders of samples submitted to the Centers for Disease Control and Prevention (CDC) Poxvirus Laboratory (Poxvirus and Rabies Branch, Division of High-Consequence Pathogens and Pathology, National Center for Emerging and Zoonotic Infectious Diseases) for routine testing. CDC reviewed viral sequencing of the samples for genomic surveillance and deemed the study as nonresearch public health surveillance. No specimen collection was performed for this study. We subjected specimens from lesion swab or crust samples of patients to DNA extraction with an EZ1 & 2 DNA tissue kit on an EZ1 Advanced XL Instrument (QIAGEN, https://www.qiagen.com). We quantified DNA concentration on a Qubit 3 Fluorometer with a dsDNA high-sensitivity assay kit (Invitrogen, https://www.thermofisher.com).

### qPCR

We performed qPCR on the DNA samples by using an Applied Biosystems 7500 Fast Dx PCR instrument with a TaqMan fast advanced master mix (Thermo Fisher Scientific, https://www.thermofisher.com) as previously described (*25*). We conducted reactions in 20-µL volumes. PCR profile was 95°C for 20 seconds, (95°C for 3 seconds, and 63°C for 30 seconds) for 40 cycles. We recorded cycle threshold (Ct) values of MPXV DNA samples.

### MPXV Amplicon-based PGS

We designed 2 pools of overlapping primers for the 10-kb region (nucleotide position 29,632 to 40,271) (Appendix Table 1) and the 15-kb region (nucleotide position 72,243 to 86,891) (Appendix Table 2) amplicons against the respective genomic region of an MPXV reference sequence (GenBank accession no. ON563414.3) by using PrimalScheme (https://primalscheme.com). We produced amplicons by using multiplex PCR with either primer pool 1 or pool 2 (10 µM). PCR profile was 98°C for 1 minute, 98°C for 20 seconds, 60°C for 30 seconds, and 72°C for 1 minute for 24 cycles (for specimens with Ct <30) or 35 cycles (Ct >30) at 72°C for 2 minutes and a 4°C hold. PCR volume was 25 µL/reaction, including 12.5 µL Q5 High-Fidelity 2X Master Mix (New England Biolabs, https://www.neb.com), 2 µL primer pool, 5 µL DNA, and 5.5 µL PCR-grade H_2_O. We verified amplicons on a 2% agarose gel by using a Gel Doc CR+ Molecular Imager (Bio-Rad Laboratories, https://www.bio-rad.com). We pooled amplicons for the 10-kb and the 15-kb regions for the same specimen together and purified them with AMPure XP beads (Beckman Coulter, https://www.beckman.com) at a volume ratio of 1:1, followed by end repairing, barcoding, and adaptor ligation with an ONT native barcoding 96 V14 kit (SQK-NBD114.96) according to the manufacturer’s protocol. We loaded DNA libraries into Flongle FLO-FLG114 flow cells and performed amplicon sequencing, base calling, and demultiplexing on an MK1C Instrument (Oxford Nanopore).

### Illumina DNA Library Sequencing

We prepared MPXV DNA libraries with an Illumina DNA prep kit and a Nextera DNA Unique Dual Indexes (Illumina, https://www.illumina.com) as described previously (*26*). We measured the concentrations of DNA libraries on the Qubit 3 Fluorometer. We determined the average sizes of libraries on a TapeStation 2400 instrument with a high-sensitivity D1000 kit (Agilent Technologies, https://www.agilent.com). We diluted DNA libraries to a concentration of 2 nM. We loaded 20 µL of the pooled libraries at a final concentration of 750 pM into the NextSeq1000 cartridge for WGS by using a P2 300-cycle kit (Illumina).

### Bioinformatic Analyses of PGS and WGS Data

#### Nanopore Data

We imported raw sequencing data into Geneious Prime 2023.2.1 (https://www.geneious.com). We aligned and mapped reads to clade IIb (GenBank accession no. ON563414.3) or clade I (GenBank accession no. KC257460) reference genomes by using Minimap2 version 2.24 (https://github.com/lh3/minimap2). We generated average sequence coverages and consensus sequences over the 10-kb and 15-kb regions of MPXV genomes per specimen. We manually identified single-nucleotide polymorphisms (SNPs) and insertions or deletions (indels) across the 2 regions in comparison with respective reference sequences. We used consensus sequences for MPXV clade and lineage assignment and phylogenetic analysis by using Nextclade 3.10.0 (https://clades.nextstrain.org).

#### Illumina Data (Metagenomic)

We processed raw data derived from Illumina sequencing through a custom-built workflow in the CLC Genomic Workbench 24.0 (QIAGEN). We extracted the mapped reads over the 10-kb and 15-kb genomic regions from the respective whole genomes. We compared sequence read depth and coverages over the 2 genomic regions derived from WGS with those from PGS.

### Nextflow Pipeline

We built a new Nextflow pipeline specific for standardizing analysis of the 10-kb and 15-kb amplicon sequences under the Nextflow Workflow Manager (version 24.04.2) with Docker and Singularity as software containers (https://github.com/CDCgov/ONT-Seq-analysis) (Appendix Figure 1). In brief, we used SEQTK version 1.4-r122 (https://github.com/lh3/seqtk) to remove adapters and low-quality calls from base called reads. We trimmed reads with Trimmomatic version 0.39 (https://github.com/usadellab/trimmomatic) and mapped them to a reference sequence (GenBank accession no. NC_063383.1) by using Minimap2 version 2.28-r1209. We refined consensus sequences generated with Ivar consensus version 1.4.3 by using ONT’s MEDAKA tool version 1.4.4. We assigned clades by using Nextclade module version 3.8.2 with additional mutation calls, phylogenetic placements, and quality checks specific for MPXV. We included custom scripts in the project repository to parse Nextclade output files, assign MPXV subclades, and summarize the resulting report into a more accessible format.

## Results

### Improved Sequence Read Depths and Coverages Using Amplicon-Based PGS Compared with WGS

**Figure 1:**
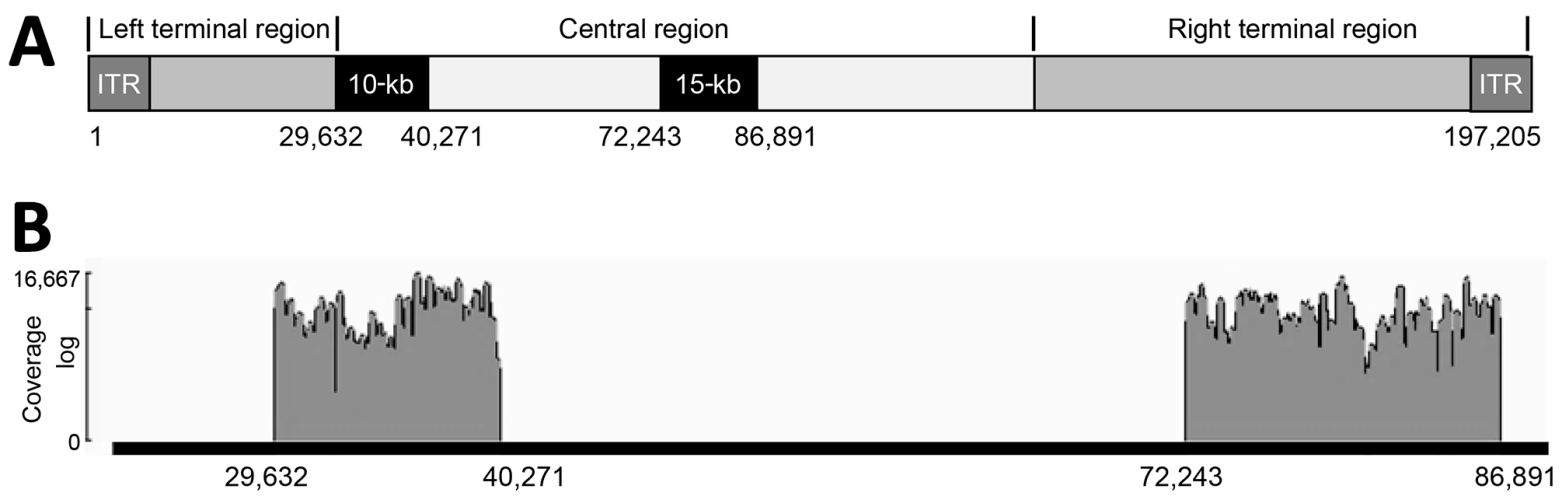
Locations and coverage of 10-kb and 15-kb amplicons of monkeypox virus for study of partial-genome amplicon sequencing for improvement of genomic surveillance during mpox outbreaks. A) Genomic positions of the 2 regions. B) Representative of sequence coverage over the 10-kb and 15-kb amplicons of monkeypox virus in clinical specimens. ITR, internal terminal repeat.

We used 2 overlapping primer pools to generate amplicons over the 10-kb or the 15-kb regions (Figure 1, panel A). Amplicon size ranged from 310 bp to 489 bp, with an average of 393 bp (Appendix Tables 1, 2). To evaluate the performance of the amplicon-based PGS approach on MPXV clinical specimens, we measured read depths and sequence coverages over the 2 genomic regions of MPXV after performing 10-kb and 15-kb PGS on MPXV DNA isolated from 36 clinical specimens (20 clade IIb, 10 clade Ia, and 6 clade Ib) with Ct <30 (Table 1). More than 93% reads produced from each specimen corresponded to MPXV (Appendix Table 3). Bioinformatic analysis demonstrated that the 10-kb and 15-kb PGS generated high-sequence read depths (Table 1) over the 2 regions of MPXV genomes (Figure 1, panel B), with an average read depth of 1,706 over the 10-kb region and 783 over the 15-kb region (Table 1). We observed complete target region coverage for all specimens (Appendix Table 3).

**Table 1:** Characteristics of MPXV specimens with Ct <30 and amplicon sequencing read depths over the 10-kb and 15-kb genomic region of MPXV for study of partial-genome amplicon sequencing for improvement of genomic surveillance during mpox outbreaks*

Specimen no.	Source/sample type	Country/year	DNA concentration, ng/μL	Ct	10-kb	15-kb
1	Back/lesion	USA/2024	4.7	19.8	1,279	554
2	Swab drag	USA/2024	5.6	22.7	840	408
3	Chest lesion	USA/2024	1.2	21.0	3,800	465
4	Lesion	USA/2024	4.3	22.6	1,455	324
5	Back/lesion	USA/2024	1.0	21.1	3,046	449
6	Leg/skin swab	USA/2024	8.4	20.1	1,625	328
7	Skin swab	USA/2024	1.0	22.0	1,417	487
8	Eye/swab drag	USA/2024	4.0	21.2	1,019	345
9	Penis/swab	USA/2024	10.7	19.2	1,097	464
10	Penis/skin swab	USA/2024	1.4	21.7	1,750	584
11	Penis/skin swab	USA/2024	9.3	19.9	2,760	479
12	Chest/skin swab	USA/2024	9.1	21.9	950	116
13	Back/skin swab	USA/2024	4.6	19.6	2,365	557
14	Hand/skin swab	USA/2024	22.0	20.5	1,870	461
15	Perianal/swab	USA/2024	2.9	21.3	1,259	432
16	Penis/skin swab	USA/2024	2.1	19.6	2,363	281
17	Perianal/swab	USA/2024	23.0	23.3	2,764	264
18	Penis/skin swab	USA/2024	1.0	20.8	1,673	652
19	Penis/lesion	USA/2024	10.9	21.5	932	241
20	Lesion	USA/2024	2.5	23.8	3,095	272
21	Crusts	DRC/2017	2.3	16.0	3,612	329
22	Vesicles	DRC/2017	1.9	16.0	1,819	1,231
23	Vesicles	DRC/2016	8.1	15.2	1,361	1,653
24	Vesicles	DRC/2017	16.0	16.7	1,421	1,607
25	Vesicles	DRC/2017	9.1	15.6	1,778	2,169
26	Crusts	DRC/2016	89.0	13.0	142	1,469
27	Vesicles	DRC/2017	62.0	18.0	797	1,056
28	Vesicles	DRC/2017	30.0	17.0	212	670
29	Vesicles	DRC/2017	28.0	22.3	1,180	762
30	Vesicles	DRC/2016	20.0	20.7	783	698
31	Swab	DRC/2024	0.8	22.6	2,022	1,520
32	Swab	DRC/2024	0.2	25.2	2,300	1,835
33	Swab	DRC/2024	6.0	17.7	1,856	1,339
34	Swab	DRC/2024	9.0	17.8	1,147	895
35	Swab	DRC/2024	0.2	25.1	1,907	1,532
36	Swab	DRC/2024	4.9	19.0	1,718	1,254

*Total DNA was extracted from MPXV specimens (n = 36) from different body sources of patients in USA in 2024 (nos. 1–20) or in DRC during 2016–2024 (nos. 21–36). DNA concentrations (ng/µL), MPXV Ct values, and read depths over the 2 genomic regions of MPXV genomes (10-kb and 15-kb) derived from amplicon sequencing on DNA samples included. Ct, cycle threshold; DRC, Democratic Republic of the Congo; MPXV, monkeypox virus; USA, United States.

To test whether the 10-kb and 15-kb PGS could still give high read depth and sequence coverage over the 2 genomic regions in specimens containing low viral DNA, we performed PGS on 8 MPXV samples: 4 clade IIb samples with Ct >30 and 4 clade Ia samples diluted with DNase-free water at a ratio of 1:1,000 or 1:10,000 to produce Ct >30 as determined by qPCR (Table 2). The amplicon-based PGS yielded high-sequence read depth across the 2 genomic regions, having an average read depth of 521 over the 10-kb region and 436 over the 15-kb region (Table 2) and >95% sequence coverage for all specimens (Appendix Table 3), even though DNA was undetectable in some specimens (Table 2). In contrast, metagenomic WGS on the same specimens produced low read depth over the 2 regions, having a maximum average read depth of 5.4 for either of the 2 regions and 12 for the whole MPXV genome (Table 2). Analysis of Ct value and coverage predicted complete target region coverage when MPXV Ct values were <31 (Appendix Table 3, Appendix Figure 2).

**Table 2:** Characteristics of MPXV specimens with Ct >30 and sequencing read depths over the 10-kb and 15-kb regions or the whole genome of MPXV for study of partial-genome amplicon sequencing for improvement of genomic surveillance during mpox outbreaks*

Specimen no. (dilution ratio)	Source/sample type	Country/year	DNA concentration, ng/μL	Ct	10-kb	15-kb	Genome
37	Penis/swab	USA/2024	14.30	35.3	587	703	10.0
38	Chin/swab	USA/2024	0.50	34.0	305	326	0.2
39	Swab	USA/2023	0.10	30.7	214	172	12.0
40	Swab	USA/2023	1.00	31.4	157	138	2.0
29 (1:1,000)	Vesicles	DRC/2017	0.02	32.3	950	310	0.4
29 (1:10,000)	Vesicles	DRC/2017	0.00	36.2	1,086	866	0.0
30 (1:1,000)	Vesicles	DRC/2016	0.02	30.0	544	613	0.0
30 (1:10,000)	Vesicles	DRC/2016	0.00	33.4	323	356	0.2

*Total DNA was extracted from MPXV specimens from different body sources of patients in USA during 2023–2024 (nos. 37–40) or in DRC during 2016–2017 (nos. 29–30). The 2 DNA samples from DRC were diluted with PCR-grade water at the ratio of 1:1,000 or 1:10,000. DNA concentrations (ng/µL), MPXV Ct values, and read depths over the 2 genomic regions (10-kb and 15-kb) derived from amplicon sequencing or over the whole genome (genome) of MPXV derived from metagenomic sequencing on DNA samples included. Ct, cycle threshold; DRC, Democratic Republic of the Congo; MPXV, monkeypox virus; USA, United States.

Next, we performed the 10-kb and 15-kb PGS on DNA isolated from formalin-fixed paraffin-embedded mpox clinical specimens, which are also challenging for WGS because of the fragmentation and crosslinking of DNA (*27*). Sequence analysis demonstrated that amplicon sequencing generated an average read depth of 792 over the 10-kb region and 694 over the 15-kb region for formalin-fixed specimens (nos. 41–45) (Table 3). Read depths were substantially lower with metagenomic WGS, which produced an average read depth of 11.2 over the whole genome (Table 3).

**Table 3:** Characteristics of formalin-fixed (nos. 41–45) or inconclusive (nos. 46–48) MPXV specimens from the United States and sequencing read depths over the 10-kb and 15-kb regions or the whole genome of MPXV for study of partial-genome amplicon sequencing for improvement of genomic surveillance during mpox outbreaks*

Specimen no.	Source	Year	DNA concentration, ng/μL	Ct	10-kb	15-kb	Genome
41	Tissue	2022	39.4	17.1	576	347	9.6
42	Tissue	2022	6.3	17.1	981	1,065	0.1
43	Rectum	2023	56.4	17.4	794	977	17.9
44	Lung	2023	3.0	30.4	869	382	0.07
45	Tissue	2024	7.4	17.3	741	698	28.4
46	Swab	2024	0.0	37.1	69	1,391	NA
47	Swab	2024	0.0	37.3	15	691	NA
48	Swab	2024	0.0	37.2	359	1,092	NA
49	Swab	2024	0.0	38.4	25	155	NA

*Total DNA was extracted from formalin-fixed MPXV tissue specimens from different body sources of patients in the United States during 2022–2024 (nos. 41–45) or inconclusive specimens (nos. 46–49) with MPXV Ct >37. DNA concentrations (ng/µL), MPXV Ct values, and read depths over the 2 genomic regions (10-kb and 15-kb) derived from amplicon sequencing or over the whole genome (genome) of MPXV derived from metagenomic sequencing on DNA samples included. Whole-genome sequencing was not performed on 3 inconclusive specimens (nos. 46–48) or failed on specimen no.49; sequencing read depths over the whole genome of MPXV were not available. Ct, cycle threshold; MPXV, monkeypox virus; NA, not available.

### Identification of Genetic Variations in the 10-kb and the 15-kb Regions of MPXV Using Amplicon-Based PGS

**Figure 2:**
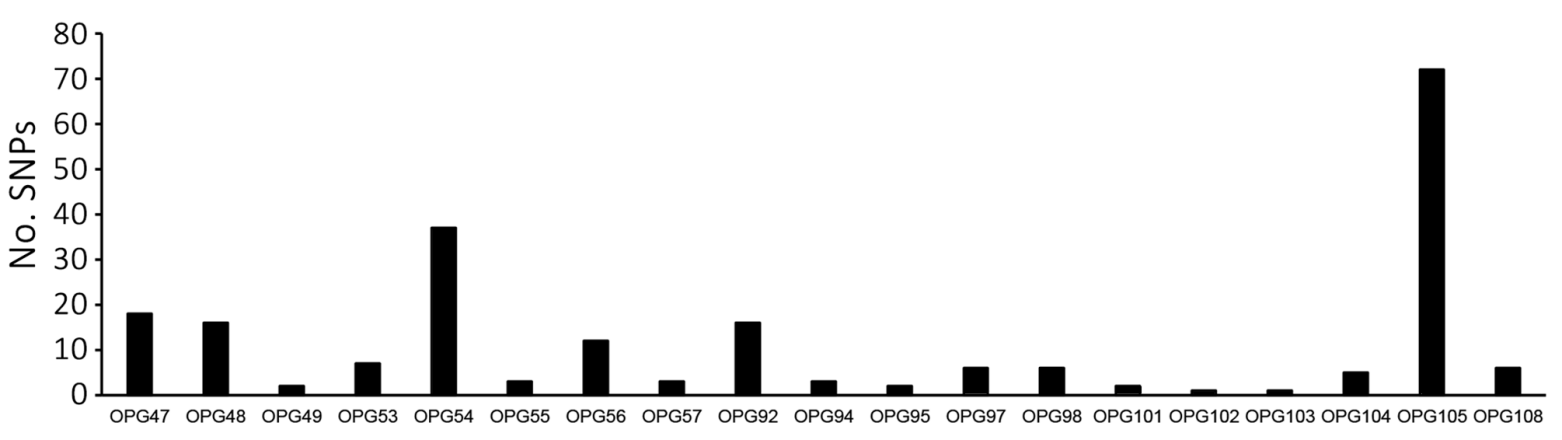
SNPs in the affected genes over the 10-kb and 15-kb genomic regions of monkeypox virus in 36 clinical specimens from United States and Democratic Republic of the Congo for study of partial-genome amplicon sequencing for improvement of genomic surveillance during mpox outbreaks. OPG, orthopoxvirus gene; SNP, single-nucleotide polymorphism.

Analyses of consensus sequences over the 10-kb and the 15-kb genomic regions (Appendix Table 4) demonstrated that amplicon-based PGS can identify many types of genetic variations in MPXV specimens from clade IIb, Ib, and Ia (Table 4; Appendix Table 5). Compared with reference sequence KC257460, the specimens showed multiple genetic variations, including 107 SNPs (28 unique) and 20 indels (2 unique) identified in 10 clade Ia specimens from DRC and 70 SNPs (13 unique) and 7 deletions (2 unique) detected in 6 clade Ib specimens from DRC (Table 4; Appendix Table 5). We detected 42 SNPs (14 unique) and 2 deletions in 20 clade IIb specimens from the United States (Table 4) compared with reference sequence ON563414.3. The deletions led to predicted amino acid changes in the encoded proteins (Appendix Table 5). Approximately 40% of the SNPs over the 10-kb and the 15-kb regions of MPXV genomes in the clade IIb specimens were GA>AA or TC>TT APOBEC3-like mutations (Table 4). However, most SNPs in the clade Ia or Ib specimens were not APOBEC3-driven mutations (Table 4), as expected when using this clade Ia reference. The SNPs and indels affected 19 structural or functional genes, including OPG047, OPG048, OPG049, OPG053, OPG054, OPG055, OPG056, and OPG057 in the 10-kb region and OPG092, OPG094, OPG095, OPG097, OPG098, OPG101, OPG102, OPG103, OPG104, OPG105, and OPG0108 in the 15-kb region (Appendix Table 5). Affected genes OPG105 (72 SNPs) and OPG054 (37 SNPs) comprised ≈50% of SNPs over the 2 genomic regions of MPXV from 36 clinical specimens (Figure 2), result that align with previous findings (*28*). Moreover, the 10-kb amplicon was able to identify a nonsynonymous SNP (C184T) and a deletion (N267del) in OPG057 gene in a specimen (no. 8) (Appendix Table 5), variations that were previously associated with tecovirimat resistance (*29*).

**Table 4:** Genetic variants over the 10-kb and 15-kb genomic regions of MPXV in 36 clinical specimens for study of partial-genome amplicon sequencing for improvement of genomic surveillance during mpox outbreaks*

Specimen no.	Country	sSNP	nsSNP	Total SNPs†	Indels	Variants‡	SNP type§	SNPs
1–20	USA	15	27	42	2	44	GA>AA	17
							Other	25
21–30	DRC	90	17	107	20	127	GA>AA	22
							Other	85
31–36	DRC	60	10	70	7	77	GA>AA	23
							Other	47

*See Table 1. DRC, Democratic Republic of the Congo; indels, insertions and deletions; MPXV, monkeypox virus; nsSNP: nonsynonymous SNP; SNP, single-nucleotide polymorphism; sSNP, synonymous SNP. USA, United States. †Total number of SNPs include sSNP and nsSNPs. ‡Variants include total number of SNPs plus indels. §SNP type GA>AA (5′–3′) includes TC>TT (5′–3′).

**Figure 3:**
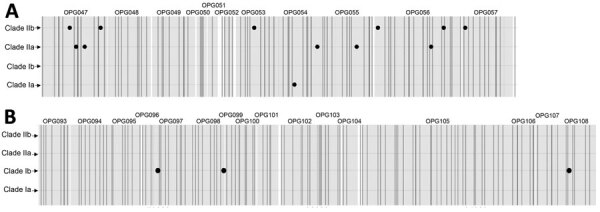
Conserved SNPs over the 10-kb and the 15-kb regions identified in silico among respective clades from 88 published moneypox virus genomes for study of partial-genome amplicon sequencing for improvement of genomic surveillance during mpox outbreaks. A) Unique SNPs over the 10-kb region. B) Unique SNPs over the 15-kb region. Large black points represent SNPs. Vertical shaded bars represent the binding sites of the amplicon primers. OPG, orthopoxvirus gene; SNP, single-nucleotide polymorphism.

In silico analysis of the 10-kb and the 15-kb regions from 88 published MPXV genome sequences revealed a pattern of conserved SNPs in different MPXV subclades (Figure 3; Appendix Table 6). Clades I and II could be separated by the 15 unique SNPs, clades Ia and Ib could be divided by the 4 SNPs, and clades IIa and IIb could be distinguished by 11 SNPs (Figure 3). This pattern of conserved SNPs indicated the potential use of the 10-kb and the 15-kb regions for differentiation of MPXV subclades.

### Correct Assignment of MPXV Subclades on the Basis of 10-kb and 15-kb Partial-Genome Sequences

**Figure 4:**
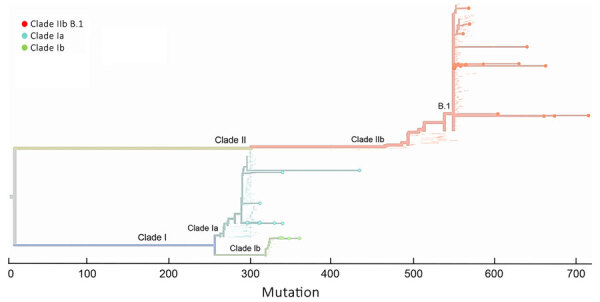
Amplicon-based phylogenetic assignment of monkeypox virus by 10-kb and 15-kb regions in Nextclade 3.10.0 (https://clades.nextstrain.org) for study of partial-genome amplicon sequencing for improvement of genomic surveillance during mpox outbreaks. Sixteen clinical specimens from Democratic Republic of the Congo were assigned to clade Ia or Ib, and 33 clinical specimens from United States were assigned to clade IIb lineage B.1.

To examine whether the 10-kb and 15-kb PGS could be used to determine MPXV subclades from clinical specimens, we imported the consensus sequences of the 2 genomic regions derived from amplicon-based PGS (Appendix Table 4) into the Nextclade web interface by using the Mpox virus (all clades) reference dataset. Clade assignment showed that MPXV in 29 specimens from the United States belonged to clade IIb lineage B. MPXV in 10 specimens from DRC were assigned to clade Ia; however, the other 6 specimens from DRC were assigned to clade Ib (Figure 4; Appendix Table 7). WGS conformed (with 100% agreement) all subclades and lineage assignments produced by the 10-kb and 15-kb PGS data (Appendix Table 7).

To increase the robustness of this evaluation, we also performed an in silico analysis of the 10-kb and the 15-kb regions extracted from 88 publicly available MPXV complete or near-complete genome sequences, including all subclades, and compared NextClade assignment of the 10-kb and the 15-kb regions to assignment using the whole-genome sequences. The partial-genome sequences were sufficient to produce correct assignments of subclade or lineages for all 88 sequences, including 25 clade Ia, 16 clade Ib, 12 clade IIa, and 35 clade IIb lineages A and B (Appendix Table 8).

To further validate the potential that the 10-kb and the 15-kb amplicons could be used to determine clade, subclade, or lineage information for inconclusive clinical specimens or specimens that had failed WGS, we selected 4 MPXV specimens (nos. 46–49) from the United States that had undetectable total DNA and MPXV Ct >37 (Table 3). For all 4 specimens, consensus sequences over the 10-kb and the 15-kb genomic regions produced from the 10-kb and 15-kb PGS successfully assigned clade IIb lineage B MPXV by Nextclade (Figure 4; Appendix Table 7).

### Automated Bioinformatic Analysis of 10-kb and 15-kb PGS Reads Using ONT Sequencing

To build a cost-free bioinformatic workflow, improve reproducibility and accessibility, and enable automatic analyses of the 10-kb and the 15-kb amplicon sequences of MPXV, we developed a Nextflow pipeline (https://github.com/CDCgov/ONT-Seq-analysis) (Appendix Figure 1). The pipeline performed quality control and reference-based assemblies of Oxford Nanopore sequencing reads and generated consensus sequences independent of clade. By using the pipeline, we verified the clade, subclade, and lineage assignments of MPXV genomes in the 49 clinical specimens, in addition to sublineage placements of 13 specimens (nos. 8–20) (Appendix Table 9).

## Discussion

Genome sequencing is at the front line of MPXV surveillance and outbreak investigation for the smallpox virus–related pathogen of high public health importance (*7*). In this study, we demonstrate that an amplicon-based PGS produces robust sequence data that can determine the clades and subclades of MPXV from clinical specimens. This high-sensitivity and low-cost PGS represents an attractive strategy for high-throughput clade typing and MPXV genomic surveillance in resource-limited settings and for specimens with low viral load.

High-quality sequences are critical for effective MPXV genomic surveillance (*30*). Clinical specimens usually contain higher human DNA background than viral DNA that can degrade under suboptimal transport or storage (*30*). Unbiased metagenomic sequencing approaches generate most non-MPXV reads or low viral-specific reads when specimens have high MPXV Ct values, causing low coverage of the whole MPXV genome, as demonstrated in this study. Several short-tiled (*31*) and long-tiled (*32**–**34*) amplicon-based WGS approaches have been developed to improve the sensitivity of MPXV WGS; however, such approaches also showed low success for samples with Ct >30, limiting potential use for specimens with low viral load. In this study, we conducted PGS by using short-tiled amplicons over the 10-kb and 15-kb regions of MPXV genome to improve PCR efficiency and lower primer dropout across the different clades. The approach selectively amplified the 2 genomic regions and yielded high read depth, even when the Ct values of MPXV specimens were >30.

Affordable MPXV genomic surveillance is critical to rapidly identify introduction of new lineages or emerging outbreaks (*35*). WGS is high-cost and resource-intensive and can be difficult for mpox-endemic regions or areas experiencing an outbreak to afford because of limited resources, capacity, or expertise. Amplicon-based WGS could be cost saving with ONT flow cells, but the number of specimens per run would be limited. In this study, we designed tiled primers that specifically target all 4 subclades of MPXV. We used the portable ONT MK1C sequencing platform with low-cost Flongle flow cells and native barcoding 96 V14 kit for PGS. One Flongle flow cell costs <$100 US and could be used for sequencing up to 40 specimens. The ONT native barcoding 96 V14 kit could be used for barcoding >288 specimens. The 10-kb and 15-kb amplicon-based PGS markedly reduced sequencing cost per specimen compared with WGS approaches. Thus, inexpensive amplicon-based PGS offers an attractive approach to complement WGS for largescale MPXV surveillance.

Bioinformatic resources and reproducibility can pose a barrier for MPXV surveillance. In this study, we described a freeware, open source, and accessible Nextflow pipeline for analyzing sequencing data produced by the 10-kb and 15-kb PGS strategy. The pipeline streamlines the entire process of amplicon sequencing data analysis, substantially reducing the need for manual intervention and the potential introduction of human errors. With detailed documentation and a feasible implementation strategy, our pipeline is suitable for varying levels of bioinformatic expertise. The pipeline’s ability to simply differentiate clades and subclades either through the Nextclade output or the SNP panel will be incredibly valuable in outbreak investigations and epidemiologic studies.

Profiling whole-genome genetic variation and phylogenetic evolution is essential for MPXV genomic surveillance. Many types of genetic variations in MPXV genomes have been identified by WGS (*23*,*36*–*39*). In this study, we used amplicon-based PGS on the 10-kb and 15-kb regions of MPXV genomes and detected numerous SNPs and indels in the 2 genomic regions of MPXV from clinical specimens. The large proportion of SNPs in MPXV lineage B genomes from clade IIb were GA>AA, consistent with previous findings based on WGS (*23*,*40*,*41*), suggesting the 10-kb and 15-kb regions might be sufficient to identify changes in APOBEC-motif mutations, an indicator of sustained human-to-human transmission of an MPXV lineage. We also found that the 10-kb and 15-kb regions possess unique conserved SNPs that can distinguish clades Ia, Ib, IIa, and IIb. Our results strongly suggest that the 10-kb and 15-kb PGS approach could produce actionable information for public health MPXV surveillance, in which a subset of samples can be submitted to WGS for more detailed analysis.

The antiviral drug tecovirimat that has been widely used to treat MPXV infections in the United States works by blocking the viral envelope protein F13 and inhibiting viral release from MPXV-infected cells (*42*,*43*). However, long-term use of the drug could induce treatment-resistant nucleotide mutations or deletions of the OPG057 gene (*29*,*42*,*44*), resulting in the spread of tecovirimat-resistant MPXV variants (*45*). WGS and targeted gene sequencing have identified numerous SNPs or indels in OPG057 associated with tecovirimat resistance (*29*,*42*,*43*). In this study, the 10-kb and 15-kb amplicon-based PGS identified tecovirimat-resistant genetic variations in the OPG057 gene that correspond to amino acid A184T mutation and N267 deletion in F13 protein (*29*) in 1 clade IIb MPXV specimen. This finding indicates that amplicon-based PGS could be useful for monitoring antiviral drug-induced resistance of MPXV. Recent clinical trials have found no substantial benefit for patients treated with tecovirimat (*46*), which might limit usefulness of genetic monitoring in some situations; however, use in severe cases is still being evaluated.

In summary, our study demonstrated that the 10-kb and 15-kb PGS procedure has the advantages of cost-effectiveness, simplicity of use, and sufficient resolution to provide information needed for public health action, such as clade assignment and identification of drug resistance. Given the outbreaks of clade Ia, Ib, and IIb mpox in the past 3 years, this 10-kb and 15-kb PGS approach offers an attractive strategy to improve overall MPXV surveillance, which can help identify importations or new outbreaks early. Its low cost and high throughput potential is especially poised for use in low-resource and outbreak settings.

Appendix Additional information about monkeypox virus partial-genome amplicon sequencing for improvement of genomic surveillance during mpox outbreaks.
