# Supplementary material for: Monkeypox Virus Partial-Genome Amplicon Sequencing for Improvement of Genomic Surveillance during Mpox Outbreaks
Source: Emerg Infect Dis. 2025 Nov;31(11):2128–37. doi: 10.3201/eid3111.250548 (PMC12704544; doi:10.3201/eid3111.250548)
Supplement: Appendix — Additional information about monkeypox virus partial-genome amplicon sequencing for improvement of genomic surveillance during mpox outbreaks. [file 25-0548-Techapp-s1.pdf]

Article DOI: https://doi.org/10.3201/eid3111.250548

EID cannot ensure accessibility for supplementary materials supplied by authors. Readers who have difficulty accessing supplementary content should contact the authors for assistance.

Monkeypox Virus Partial-Genome Amplicon Sequencing for Improvement of Genomic Surveillance during Mpox Outbreaks

Appendix

Appendix Table 1. Primers for the 10-kb amplicon (reference: ON563414.3)

Primer Number	Pool	Primer sequence (5'-3')	Genome position	Amplicon size
46_RIGHT	Pool 2	GCCGATGATATTCTAGAATCGTTGA	40247-40271	445
46_LEFT	Pool 2	CATCTTTCATCATCAGACACCCAA	39827-39850	
45_RIGHT	Pool 1	TCTGGGAGAGCTACAAAGTCACT	39921-39943	369
45_LEFT	Pool 1	CCCAATAGGTGTTCTGGAGAATCAG	39575-39599	
44_RIGHT	Pool 2	GGTTGAATTTATGCTCTGCGGC	39662-39683	360
44_LEFT	Pool 2	ACAACGCATCTAGACTTCTGGC	39324-39345	
43_RIGHT	Pool 1	TAGTTCCCACTACACGTGTCGA	39470-39491	377
43_LEFT	Pool 1	GTGGCTAGATACCCAATCTCTTTCA	39115-39139	
42_RIGHT	Pool 2	ACTTCGGCAAATTTCGACGGA	39217-39237	390
42_LEFT	Pool 2	TGTGCAATGGGTCAATCCTATAACT	38848-38872	
41_RIGHT	Pool 1	AGAACGCCATCATGTTAAACAGGA	39039-39062	380
41_LEFT	Pool 1	ACCGTATCGTTCTAAAGCCAGTG	38683-38705	
40_RIGHT	Pool 2	TCGCCTCCAATTCCTATGTCTAGA	38812-38835	418
40_LEFT	Pool 2	GCGCCCACAATTCATCTCTAGA	38418-38439	
39_RIGHT	Pool 1	GGCTGTCGTCAAAAGGTTTAGC	38624-38645	340
39_LEFT	Pool 1	TTACCCGGGTACGTGGATATCA	38306-38327	
38_RIGHT	Pool 2	TGCCGTATCAGTTTATGGAGCC	38356-38377	374
38_LEFT	Pool 2	AATCAAATGGTCGGCTCTCTGG	38004-38025	
37_RIGHT	Pool 1	GACTAGTACACGAACTATCATTCTAGTAGG	38207-38236	338
37_LEFT	Pool 1	ACGCTCCTGATTGTGTTTCGAA	37899-37920	
36_RIGHT	Pool 2	TTCGAAACACAATCAGGAGCGT	37899-37920	342
36_LEFT	Pool 2	AGGTACCGTGACTTTACACAACG	37579-37601	
35_RIGHT	Pool 1	TCCGAGAATACTGGAATGGGGA	37794-37815	367
35_LEFT	Pool 1	GTAAAGCCCCTAGTTCGACTATCG	37449-37472	
34_RIGHT	Pool 2	CGTCCGTCGATATTGAAACTGC	37490-37511	432
34_LEFT	Pool 2	CAATGGAATGCAAAACCCCATGG	37080-37102	
33_RIGHT	Pool 1	GACTTGAACGCAGTCACAACAAA	37364-37386	372
33_LEFT	Pool 1	GGAGTAGGACGTCTAGCCAGTAT	37015-37037	
32_RIGHT	Pool 2	TTCCATACTGGCTAGACGTCCT	37020-37041	421
32_LEFT	Pool 2	AATGTAAAGCCCTGAAACCCCC	36621-36642	
31_RIGHT	Pool 1	AGCATCTTCGTATCCACCATGC	36902-36923	404
31_LEFT	Pool 1	GCTTCTCCTCCTATTTGTGGAAGAA	36520-36544	
30_RIGHT	Pool 2	TCGGTCCAATATCTGTCAAGGAGA	36580-36603	415
30_LEFT	Pool 2	CTTGGCTGTCTCATTCAATAGGGT	36189-36212	
29_RIGHT	Pool 1	GATAACGCCAAAATCGCTGCTC	36419-36440	429
29_LEFT	Pool 1	ACAAAAAGCCCCAATTTACAAACGA	36012-36045	
28_RIGHT	Pool 2	TGTGGAGGTGTTAAAAACAGAGACA	36083-36107	383
28_LEFT	Pool 2	CTCCTGTAGATATGTGATAAAAATCGTCG	35725-35753	
27_RIGHT	Pool 1	GTGTTGCCAATGATTCATCCCC	35988-36009	382
27_LEFT	Pool 1	GTAAACTCCGCCGTAGTTTCCA	35628-35649	
26_RIGHT	Pool 2	TCGAGGCCACAAAATTATATAGTCCC	35651-35676	371
26_LEFT	Pool 2	TTCGAAAAAGTGAAACATGCGGTT	35306-35329	
25_RIGHT	Pool 1	CGTGGGCCATGGGATTAAACTA	35547-35568	354
25_LEFT	Pool 1	ACTCGGTATCTACTTTATCTGCCGA	35215-35239	
24_RIGHT	Pool 2	TTTTCCCCTAGCACTGTATTCGG	35236-35258	360
24_LEFT	Pool 2	TGGAAATCGTAATACCAGTTATGTTCAAC	34899-34927	
23_RIGHT	Pool 1	CGAACTACCCTGTTGTGACAACT	35107-35129	433

Primer Number	Pool	Primer sequence (5'-3')	Genome position	Amplicon size
23_LEFT	Pool 1	GTTTCTGCCATTTATCCAGTCTGAG	34697-34721	
22_RIGHT	Pool 2	TCATGTGTACCAAAACAGACTGTGA	34794-34818	448
22_LEFT	Pool 2	CCTCCGCATACGCATTACAGTT	34371-34392	
21_RIGHT	Pool 1	CTATCCCTACTAATGTCACTTGTGCT	34606-34631	364
21_LEFT	Pool 1	GCTTCCGCCGATCCTGTATTAA	34268-34289	
20_RIGHT	Pool 2	TATCGGAGAATGTTCGGCTCCT	34318-34339	359
20_LEFT	Pool 2	CGACTGTCGTGTTTGCGTTTAG	33981-34002	
19_RIGHT	Pool 1	TCCGTGGTTTCTAGTGGGTGTA	34162-34183	380
19_LEFT	Pool 1	TGAGATGATATACTCTATTTTGGTGGAGG	33804-33832	
18_RIGHT	Pool 2	TGAAGAAATTGCAAAATATGGACACTCA	33905-33932	397
18_LEFT	Pool 2	CCAAATTCTACACGTGCTTCGG	33536-33557	
17_RIGHT	Pool 1	CAATGTGAACTTTTTACTTTCTTACCGG	33751-33778	480
17_LEFT	Pool 1	GTACCTCATCAACCGGTAAAAGACT	33299-33323	
16_RIGHT	Pool 2	TCTCGAGTTATGATGATAGAAGAATCCG	33326-33353	358
16_LEFT	Pool 2	TCCTCTGTGGCTAGCGCTATAA	32996-33017	
15_RIGHT	Pool 1	ACCATGGATTTGACTGTGTGGAG	33270-33292	389
15_LEFT	Pool 1	CAGAATCTTCGTACCTCAAACTCGA	32904-32928	
14_RIGHT	Pool 2	TCGAGTTTGAGGTACGAAGATTCTG	32904-32928	349
14_LEFT	Pool 2	ACGTCGAGCTAATTTCTAACGCT	32580-32602	
13_RIGHT	Pool 1	TGACTTGACTGGTAGAGTGCGT	32800-32821	371
13_LEFT	Pool 1	TTAACGCCTCCCATTCGGATTC	32451-32472	
12_RIGHT	Pool 2	TGATAATGCATCTTCCGTGCCC	32422-32443	366
12_LEFT	Pool 2	GTCCAAAATGATGCCTCTGCCT	32078-32099	
11_RIGHT	Pool 1	CGATTATTCAACACCCATGAATGTCG	32331-32356	448
11_LEFT	Pool 1	TCTAGCCTCGGTAATCTGCACT	31909-31930	
10_RIGHT	Pool 2	TTTGCAGCCAGTGACGGAATAG	31965-31986	325
10_LEFT	Pool 2	TGGAAGCGAATGATCCGGAAAA	31662-31683	
9_RIGHT	Pool 1	ACGGATTTCAGATGGCCATTGA	31880-31901	450
9_LEFT	Pool 1	TGAGTTTAACTGGAAGAGCCACAG	31452-31475	
8_RIGHT	Pool 2	CCACCGAGTGAAGAAACCGTTA	31521-31542	402
8_LEFT	Pool 2	GTTTTGAATTTCTTAGTCGTGTACAATGC	31141-31169	
7_RIGHT	Pool 1	TCGTCGCAGATAGATTGATTTCTGA	31388-31412	406
7_LEFT	Pool 1	GGTCTAGACAAACCCTCGTAACTG	31007-31030	
6_RIGHT	Pool 2	CAGTTACGAGGGTTTGTCTAGACC	31007-31030	410
6_LEFT	Pool 2	TCTTCGCCTCATATACCACTTAATGAC	30621-30647	
5_RIGHT	Pool 1	ACGTCGTCAATTTATTACGTGCTTC	30913-30937	386
5_LEFT	Pool 1	ACCTCTGGGAGAAAGATAATTTGACC	30552-30577	
4_RIGHT	Pool 2	GGTCAAATTATCTTTCTCCCAGAGGT	30552-30577	359
4_LEFT	Pool 2	TGTGGGATTTGGTAGACCTCCT	30219-30240	
3_RIGHT	Pool 1	ACCACGCAAATCATACAAGTATCCA	30471-30495	364
3_LEFT	Pool 1	GGATGCCACTGCTGGATTACAT	30132-30153	
2_RIGHT	Pool 2	ATGTAATCCAGCAGTGGCATCC	30132-30153	388
2_LEFT	Pool 2	CCATTCCATTCCATCCCATATATTCCA	29766-29792	
1_RIGHT	Pool 1	ATGATCAGTGGCAGTTTGGACC	30037-30058	427
1_LEFT	Pool 1	ACAAATCATATCCGGCAGCACC	29632-29653	

Appendix Table 2. Primers for the 15-kb amplicon (reference: ON563414.3)

Primer number	Pool	Primer sequence (5'--3')	Genome position	Amplicon size
64_RIGHT	Pool 2	TGTGTTTATACAATGGACTGGAGGAA	86866 - 86891	408
64_LEFT	Pool 2	ATGTTCAGCGCCGTAGTAACTC	86484 - 86505	
63_RIGHT	Pool 1	TCGCTAGACATTTAGCCCTATGG	86762 - 86784	468
63_LEFT	Pool 1	GTTTCATGGTTTCGAAGCGGTG	86317 - 86338	
62_RIGHT	Pool 2	TCTGGAGGTCTATCATCGGGATTT	86441 - 86464	402
62_LEFT	Pool 2	CCGCATTGGTGTTCCGATCTT	86063 - 86083	
61_RIGHT	Pool 1	GGAACGGTAGCTGCTAAACGTT	86275 - 86296	384
61_LEFT	Pool 1	GGAGACGCGTGGATGGTTAAAA	85913 - 85934	
60_RIGHT	Pool 2	TTTTAACCATCCACGCGTCTCC	85913 - 85934	381
60_LEFT	Pool 2	ACGCATGCAATTTAGAATACGTTAGAG	85554 - 85580	
59_RIGHT	Pool 1	ATCATCCTTACCAGCGGCTAGA	85795 - 85816	373
59_LEFT	Pool 1	GGGAGATTTGGCTCTGTGCATA	85444 - 85465	
58_RIGHT	Pool 2	ACTGGAGATATGCACAGAGCCA	85452 - 85473	315
58_LEFT	Pool 2	AGCCCCGCTTCTATTTACTCCT	85159 - 85180	
57_RIGHT	Pool 1	TGGATGCACCATCATCTGAAGTT	85366 - 85388	340
57_LEFT	Pool 1	GGATTTTCCACAAATGCGCCTC	85049 - 85070	
56_RIGHT	Pool 2	GAGGCGCATTTGTGGAAAATCC	85049 - 85070	389
56_LEFT	Pool 2	GCCTTGTGATCTTCTCGCTAGTT	84682 - 84704	
55_RIGHT	Pool 1	TCAGATAGTTTCCTTTCCATTCTCATGA	84924 - 84951	389
55_LEFT	Pool 1	GTCAACGTGTATCCTGGAGTATGG	84563 - 84586	
54_RIGHT	Pool 2	GAGCGGCCTCAATACCAAAGAT	84599 - 84620	402
54_LEFT	Pool 2	ACATCAAGAGAGCAGAAATAACCGA	84219 - 84243	
53_RIGHT	Pool 1	CCCGTATAGTCAGAGATAGGAATCTTG	84445 - 84471	489
53_LEFT	Pool 1	CACTACTGAAAAGAGTGGTGCTGT	83983 - 84006	
52_RIGHT	Pool 2	GTTTCTTTTCGAAGAGTGATGTCTGG	84155 - 84180	441
52_LEFT	Pool 2	CGTCATTGATGATGTGAGAGAGAAATAC	83740 - 83767	
51_RIGHT	Pool 1	CTGGACAGGGCTTGTTGTGTAA	83955 - 83976	429
51_LEFT	Pool 1	TCCAGATGAGTCCATGACTTGGT	83548 - 83570	
50_RIGHT	Pool 2	CTCAGTGGTGGGTTTGACGAAT	83674 - 83695	310
50_LEFT	Pool 2	AACATCACGTACCGGAACACTG	83386 - 83407	
49_RIGHT	Pool 1	GCTGCGTACTTGATGAGCGT	83475 - 83496	370
49_LEFT	Pool 1	TGAAAATGGCCAAAGCGGGT	83127 - 83146	
48_RIGHT	Pool 2	ACCTCTTCCTTCTGGATCCTTAGA	83266 - 83290	408
48_LEFT	Pool 2	CTGAGACCAAATTCGACGTTCA	82883 - 82905	
47_RIGHT	Pool 1	ATGGATTCCAAGTAGTCCGC	83021 - 83042	454
47_LEFT	Pool 1	TTGGGGAAATATGGAAGAGAGTT	82589 - 82613	
46_RIGHT	Pool 2	CTTTGAATGTCACCCCAAAAC	82858 - 82880	394
46_LEFT	Pool 2	ATTCATGGAGCCCCCGTTTAT	82487 - 82508	
45_RIGHT	Pool 1	CATAAACGGGGGCTCCATGA	82487 - 82508	338
45_LEFT	Pool 1	CCAATTAACAAGAATACGTCAAGGA	82171 - 82199	
44_RIGHT	Pool 2	ACGGCTTTAGGATTTTGCTCC	82416 - 82437	444
44_LEFT	Pool 2	GACCGCTAGATCCGTAATTGGT	81994 - 82016	
43_RIGHT	Pool 1	TGGGCATTCCTACCTCATTAAC	82033 - 82055	334
43_LEFT	Pool 1	TACTTTCCCATCCCTCCGTTGA	81722 - 81743	
42_RIGHT	Pool 2	GCCGAGCGACGATATAACTTC	81962 - 81983	449
42_LEFT	Pool 2	GAACAGCGAATGTATGCAACC	81535 - 81556	
41_RIGHT	Pool 1	ACGTTAATATCATCCAACTTGTTGACG	81595 - 81621	357
41_LEFT	Pool 1	AGGTGCTATGGATGGGGCATTA	81265 - 81286	
40_RIGHT	Pool 2	CTCCTAAGAGCGTGTACCGTAAC	81476 - 81497	409
40_LEFT	Pool 2	AGGGAAATTTGATTGTATACCTTCGGT	81089 - 81115	
39_RIGHT	Pool 1	ACGTAACCTTAGAGATTACAGCCA	81142 - 81167	423
39_LEFT	Pool 1	TCTCGGTAGCTACTCGTTTGGA	80745 - 80766	
38_RIGHT	Pool 2	ATGTCATGACGGACGAACAAA	81014 - 81038	477
38_LEFT	Pool 2	ATTAATCAATTAGTAGAGATGAGATAAGAACA	80562 - 80591	
37_RIGHT	Pool 1	TCCGATTGGATTACTAGCGTTAGC	80660 - 80683	404
37_LEFT	Pool 1	TCAAAAAGGTCATAACAAGTTTCCTGT	80280 - 80307	
36_RIGHT	Pool 2	TCGGCTATCTCTACTCCAGTTA	80524 - 80547	446
36_LEFT	Pool 2	AGAAACGTACTACTGAGATATACTACCGA	80102 - 80130	
35_RIGHT	Pool 1	ACCGGAGTATAATTTATGGATGATTCGT	80211 -80238	424
35_LEFT	Pool 1	CCTTAATAAGATAGTCCGCAACAAAGTAG	79815 -79843	
34_RIGHT	Pool 2	CGCGTACGGATCTCTTGCTATT	80071 - 80092	464
34_LEFT	Pool 2	AGATGCCCGTTTCCAGATCAA	79629 - 79650	
33_RIGHT	Pool 1	AGCGTCTGATTTGGTAACTCGA	79766 - 79788	401
33_LEFT	Pool 1	GCCGCCATCATGATCCTATTCT	79388 - 79409	
32_RIGHT	Pool 2	CTAGACGCCACGGGGTTTAA	79598 - 79610	439
32_LEFT	Pool 2	CAGAAAGTGCAAATGAGGTCGC	79172 - 79193	
31_RIGHT	Pool 1	GTGGCACCATCTAATATACCGT	79271 - 79292	461
31_LEFT	Pool 1	TGGGACATTTCAACGTAGACC	78832 - 78853	

Primer number	Pool	Primer sequence (5'--3')	Genome position	Amplicon size
30_RIGHT	Pool 2	CGGTTTATCTAACGACACAACATC	79104 - 79128	384
30_LEFT	Pool 2	AGATGAAGGACAGTTCTTTCCAGA	78745 - 78769	
29_RIGHT	Pool 1	TCGAGCGCGGCTACTATAACTA	78810 - 78831	414
29_LEFT	Pool 1	CCTCGTCGTGATATCGCATTTTC	78418 - 78440	
28_RIGHT	Pool 2	TGTCCATAGTCCCGTTCCGTAT	78637 - 78658	444
28_LEFT	Pool 2	CTCTTTCAGAGGAATACAGACAATTGAC	78215 - 78242	
27_RIGHT	Pool 1	AGGATCTATTGCGGTGGTAGCT	78381 - 78402	354
27_LEFT	Pool 1	ACGATGTTCTTCGCAGATGATGA	78049 - 78071	
26_RIGHT	Pool 2	TCATAACAATGACCCACAGCTTCT	78212 - 78189	341
26_LEFT	Pool 2	GCCTAAACGAAAAATACCCGATCC	77849 - 77872	
25_RIGHT	Pool 1	AACGCAGATGTTTGAGTTGTTATCC	77941 - 77965	395
25_LEFT	Pool 1	TCTAGCCCTCTTCAAGAACCCA	77571 - 77592	
24_RIGHT	Pool 2	AAAGAATGTTTAAACGTGGGCTCT	77746 - 77723	316
24_LEFT	Pool 2	ACCTATCGGAGACAACAAGGCT	77408 - 77429	
23_RIGHT	Pool 1	TCTCATGGACGTGGAAGGAGAA	77471 - 77492	383
23_LEFT	Pool 1	TCACAGGGGCAATGTTTACCAC	77110 - 77131	
22_RIGHT	Pool 2	CAAGATAGGTGATGATCCTTGACGG	77312 - 77336	406
22_LEFT	Pool 2	AGAAAACCTCATCGAAGAAGATACCA	76931 - 76956	
21_RIGHT	Pool 1	GGAAATTTGGATTTTGCACCAGC	77029 - 77007	331
21_LEFT	Pool 1	TTCCTTCTCGTAGGCACACAATC	76677 - 76699	
20_RIGHT	Pool 2	TGAATACCCGTACCGATGTTACAAA	76867 - 76891	339
20_LEFT	Pool 2	GAGGAAATCCAACCGCCTTTCT	76553 - 76574	
19_RIGHT	Pool 1	GCTGGTCAGAACTATCGTCGTT	76585 - 76606	354
19_LEFT	Pool 1	TTGTTCTATAGAGCCGGTTGCC	76253 - 76274	
18_RIGHT	Pool 2	AGTTGTGAGATTAACCCCGCAAG	76463 - 76485	421
18_LEFT	Pool 2	CAACCTATTTACTGGTGCTCCTGT	76065 - 76088	
17_RIGHT	Pool 1	AGCCCGTAGAGGATATGAACACT	76203 - 76181	363
17_LEFT	Pool 1	GGTGGCGCTAGTCATCACATTA	75819 - 75840	
16_RIGHT	Pool 2	GGTACACTGGTATATGTGCCCAAA	76032 - 76055	453
16_LEFT	Pool 2	CATGGAAGTTATCGCTGATCGTCT	75603 - 75626	
15_RIGHT	Pool 1	CAGGACATTGATGCTCTAGACCG	75684 - 75706	365
15_LEFT	Pool 1	GCACCTAGACAAGTTGCTGGTA	75342 - 75363	
14_RIGHT	Pool 2	TCCGTGGTAGCAATAATCATCGG	75537 - 75559	397
14_LEFT	Pool 2	CTTGTAATTCTAGCGCTGTTGTCG	75163 - 75186	
13_RIGHT	Pool 1	AATTTGTTGGAGATCCTGGGGC	75234 - 75255	391
13_LEFT	Pool 1	ACACTCAGTGAACGTATCTCGTC	74856 - 74878	
12_RIGHT	Pool 2	CTCGTCTGAATGTTTAACGCAGC	75096 - 75118	424
12_LEFT	Pool 2	CTACTGTTCCCGTCAATAGAGCAA	74695 - 74718	
11_RIGHT	Pool 1	AGTCGTCTGTATGCTTGCTGC	74829 - 74849	321
11_LEFT	Pool 1	GATCCAGAGTGTTCGAATGCCA	74529 - 74550	
10_RIGHT	Pool 2	TCCATCTGTAAGTTATTCACGCTCG	74617 - 74641	371
10_LEFT	Pool 2	ACTGTAGAGGGTCGACAATCGT	74271 - 74292	
9_RIGHT	Pool 1	CCGCCGACTGTTGTCTTAGAAT	74418 - 74439	381
9_LEFT	Pool 1	GCTGTCGCACTTTTAATCGTACTC	74059 - 74082	
8_RIGHT	Pool 2	CATCGCATTTTCCAGTGTCGTG	74160 - 74181	470
8_LEFT	Pool 2	GGAATGTTGTTTTCGGATAGAGTTCAA	73712 - 73738	
7_RIGHT	Pool 1	AATAATGCCGAAAGACCACCGG	74005 - 74026	401
7_LEFT	Pool 1	CGTCTCTCAACAAACTACGAGGA	73626 - 73648	
6_RIGHT	Pool 2	TCCTCGTAGTTTGTTGAGAGACG	73626 - 73648	331
6_LEFT	Pool 2	CGATTGTAACAAGGAACTGATGCC	73318 - 73341	
5_RIGHT	Pool 1	ATTCCCTGGAAAACGCATCAGT	73548 - 73568	456
5_LEFT	Pool 1	CCTTGGGCGGATCTGTAAACAT	73113 - 73134	
4_RIGHT	Pool 2	AATATTTTCGGCTCCGCGGTAG	73168 - 73189	378
4_LEFT	Pool 2	CACTGATTCCTTTTTCGATGCCG	72812 - 72834	
3_RIGHT	Pool 1	AGACACTTCTTCGCTGAACTCTG	73047 - 73069	384
3_LEFT	Pool 1	TCTCCAGAAATCTGTGGCGTTG	72686 - 72707	
2_RIGHT	Pool 2	CAGTTCATGTATGCAACGCCAC	72699 - 72720	373
2_LEFT	Pool 2	TCCATTAGCCTTTCCACTTCTGC	72348 - 72370	
1_RIGHT	Pool 1	AGCCCACTATTCTAGCCACACT	72609 - 72630	388
1_LEFT	Pool 1	TTTCAAGCCGGCTATATATTCTGCT	72243 - 72270	

Appendix Table 3. Amplicon sequence reads and coverages over the 10-kb and the 15-kb genomic regions of MPXV.

Specimens	Total reads	MPXV-mapped reads (%)	10-kb coverage (%)	15-kb coverage (%)
1	46033	94.7	100	100
2	28496	94.8	100	100
3	82929	94.9	100	100
4	40209	94.9	100	100
5	71747	95.0	100	100
6	43145	95.1	100	100
7	38959	95.3	100	100
8	34568	94.9	100	100
9	40794	95.1	100	100
10	50147	95.1	100	100
11	73886	95.0	100	100
12	22368	95.3	100	100
13	25670	95.0	100	100
14	50513	95.1	100	100
15	37550	94.8	100	100
16	50622	94.8	100	100
17	59691	95.1	100	100
18	52731	94.6	100	100
19	25720	93.6	100	100
20	71232	95.0	100	100
21	86555	94.4	100	100
22	70256	94.1	100	100
23	73336	93.7	100	100
24	75591	94.0	100	100
25	98930	94.0	100	100
26	21715	93.0	100	100
27	21140	93.2	100	100
28	26437	93.4	100	100
29	13150	93.4	100	100
30	25899	93.2	100	100
31	89259	94.1	100	100
32	102872	94.3	100	100
33	79300	94.1	100	100
34	51770	94.2	100	100
35	85456	94.4	100	100
36	73919	94.0	100	100
37	71052	93.8	95.2	98.5
38	112674	93.6	96.1	100
39	104912	85.3	99.8	100
40	50497	85.2	99.9	100
29 (1:1000)	51133	92.6	100	100
29 (1:10000)	47035	92.7	100	100
30 (1:1000)	26190	92.4	100	100
30 (1:10000)	16446	92.1	100	100
41	43115	93.1	100	100
42	27960	93.3	100	100
43	20817	93.3	100	99.8
44	36807	92.9	100	100
45	52042	93.1	100	100
46	25949	93.4	83.0	90.8
47	11428	93.5	92.0	81.6
48	21420	94.0	85.1	90.7
49	1029	92.1	84.1	96.6

Appendix Table 4. GenBank accession of amplicon consensus sequences of the 10-kb and the 15-kb genomic regions of MPXV in the 49 specimens.

Specimen No.	GenBank 10-kb	accession 15-kb	Specimen No.	GenBank 10-kb	accession 15-kb
1	PX122318	PX122319	26	PX123349	PX123350
2	PX122280	PX122281	27	PX123337	PX123338
3	PX122292	PX122293	28	PX123345	PX123346
4	PX122316	PX122317	29	PX123347	PX123348
5	PX122326	PX122327	30	PX123331	PX123332
6	PX122274	PX122275	31	PX123351	PX123352
7	PX122276	PX122277	32	PX123361	PX123362
8	PX122272	PX122273	33	PX123353	PX123354
9	PX122294	PX122295	34	PX123355	PX123356
10	PX122296	PX122297	35	PX123359	PX123360
11	PX122298	PX122299	36	PX123357	PX123358
12	PX122300	PX122301	37	PX122324	PX122325
13	PX122302	PX122303	38	PX122278	PX122279
14	PX122304	PX122305	39	PX122262	PX122263
15	PX122306	PX122307	40	PX122264	PX122265
16	PX122308	PX122309	41	PX122320	PX122321
17	PX122310	PX122311	42	PX122314	PX122315
18	PX122312	PX122313	43	PX122266	PX122267
19	PX122268	PX122269	44	PX122290	PX122291
20	PX122270	PX122271	45	PX122282	PX122283
21	PX123335	PX123336	46	PX122284	PX122285
22	PX123343	PX123344	47	PX122286	PX122287
23	PX123333	PX123334	48	PX122322	PX122323
24	PX123339	PX123340	49	PX122288	PX122289
25	PX123341	PX123342			

Appendix Table 5. MPXV genomic variation over the 10-kb and the 15-kb regions in the clinical specimens (n=36) (Reference: ON563414.3)*

Specimen No.	10-kb genomic position	SNPs	AA change	Insertion	Deletion	Gene
3	38,802	TC>TT	D 84 N			OPG056
8			N241/-		32,443-32,445	OPG049
8			N267/-		39,411-39,413	OPG057
8	39,663	C>T	A 184 T			OPG057
9	34,294	TC>TT				OPG053
10	37,042	GA>AA	S 20 F			OPG055
11	34,294	TC>TT				OPG053
12	37,042	GA>AA	S 20 F			OPG055
13	37,042	GA>AA	S 20 F			OPG055
14	34,294	TC>TT				OPG053
15	34,294	TC>TT				OPG053
16	34,294	TC>TT				OPG053
17	34,294	TC>TT				OPG053
18	34,294	TC>TT				OPG053
18	37,484	TC>TT				OPG056
19	35,149	GA>AA				OPG054
20	35,149	GA>AA				OPG054
Specimen No.	15-kb genomic position	SNPs	AA change	Insertion	Deletion	Gene
1	84,681	A>T	Q 1180 L			OPG105
2	84,681	A>T	Q 1180 L			OPG105
3	80,548	T>C				OPG103
3	83,770	TC>TT				OPG105
3	84,681	A>T	Q 1180 L			OPG105
4	84,681	A>T	Q 1180 L			OPG105
5	80,933	G>T				OPG104
5	84,681	A>T	Q 1180 L			OPG105
6	84,681	A>T	Q 1180 L			OPG105
7	84,681	A>T	Q 1180 L			OPG105
8	82,480	A>G				OPG105
8	84,681	A>T	Q 1180 L			OPG105
9	84,681	A>T	Q 1180 L			OPG105
10	84,681	A>T	Q 1180 L			OPG105
11	84,681	A>T	Q 1180 L			OPG105
12	84,681	A>T	Q 1180 L			OPG105
13	72,297	TC>TT	R 223 K			OPG092
13	84,681	A>T	Q 1180 L			OPG105
14	84,681	A>T	Q 1180 L			OPG105
15	84,681	A>T	Q 1180 L			OPG105
16	84,681	A>T	Q 1180 L			OPG105
17	84,681	A>T	Q 1180 L			OPG105
18	79,151	A>T				OPG102
18	84,681	A>T	Q 1180 L			OPG105
19	84,681	A>T	Q 1180 L			OPG105
19	86,737	TC>TT	E 114 K			OPG105
20	84,681	A>T	Q 1180 L			OPG105
	Ref. KC257460					
Specimen No.	10-kb genomic position	SNPs	AA change	Insertion	Deletion	Gene
21	31,702		Yes	31,702		OPG047
21	33,092	A>G				OPG047
21	33,159	G>A				OPG048
21	37,010	G>A				OPG054
21	37,556	G>A				OPG054
21	39,173	G>T	T 602 N			OPG056
22	31,702		Yes	31,702		OPG047
22	33,092	A>G				OPG047
22	33,159	G>A				OPG048
22	34,192	G>A	A 328 V			OPG049
22	37,010	G>A				OPG054
22	37,556	GA>AA				OPG054
22	39,173	G>T	T 602 N			OPG056
23	31,702		Yes	31,702		OPG047
23	33,092	A>G				OPG047
23	33,159	G>A				OPG048
23	37,010	G>A				OPG054
23	37,556	GA>AA				OPG054
23	39,173	G>T	T 602 N			OPG056
24	31,702		Yes	31,702		OPG047

Specimen No.	10-kb genomic position	SNPs	AA change	Insertion	Deletion	Gene
24	33,092	A>G				OPG047
24	33,159	G>A				OPG048
24	37,010	G>A				OPG054
24	37,556	GA>AA				OPG054
24	39,173	G>T	T 602 N			OPG056
24	41,178	G>A				OPG057
25	31,702		Yes	31,702		OPG047
25	33,159	G>A				OPG048
25	37,010	G>A				OPG054
25	37,556	GA>AA				OPG054
25	39,173	G>T	T 602 N			OPG056
25	41,178	G>A				OPG057
26	31,702		Yes	31,702		OPG047
26	33,092	A>G				OPG047
26	33,159	G>A				OPG048
26	36,923	C>T				OPG054
26	37,010	G>A				OPG054
26	37,556	GA>AA				OPG054
26	37,580	A>G				OPG054
26	39,173	G>T	T 602 N			OPG056
27	31,702		Yes	31,702		OPG047
27	33,092	A>G				OPG047
27	33,159	G>A				OPG048
27	37,010	G>A				OPG054
27	37,556	GA>AA				OPG054
27	39,173	G>T	T 602 N			OPG056
28	31,702		Yes	31,702		OPG047
28	33,159	G>A				OPG048
28	34,192	G>A	A 328 V			OPG049
28	36,628	C>T				OPG054
28	37,010	G>A				OPG054
28	37,556	GA>AA				OPG054
28	39,173	G>T	T 602 N			OPG056
29	31,702		Yes	31,702		OPG047
29	33,159	G>A				OPG048
29	37,010	G>A				OPG054
29	37,556	GA>AA				OPG054
29	39,173	G>T	T 602 N			OPG056
30	31,702		Yes	31,702		OPG047
30	33,092	A>G				OPG047
30	33,159	G>A				OPG048
30	37,010	G>A				OPG054
30	37,556	GA>AA				OPG054
30	39,173	G>T	T 602 N			OPG056
31	31,701	T>C	E481G			OPG047
31	33,468	C>T				OPG048
31	37,295	A>C				OPG054
31	37,556	GA>AA				OPG054
32	31,701	T>C	E481G			OPG047
32	33,092	A>G				OPG047
32	33,468	CT>TT				OPG048
32	37,295	A>C				OPG054
32	37,556	GA>AA				OPG054
32	38,545		yes		38,545 (161P-)	OPG055
33	31,701	T>C	E481G			OPG047
33	33,092	A>G				OPG047
33	33,468	CT>TT				OPG048
33	37,295	A>C				OPG054
33	37,556	GA>AA				OPG054
34	31,701	T>C	E481G			OPG047
34	33,092	A>G				OPG047
34	33,468	TC>TT				OPG048
34	37,295	A>C				OPG054
34	37,556	GA>AA				OPG054
35	31,701	T>C	E481G			OPG047
35	33,092	A>G				OPG047
35	33,468	TC>TT				OPG048
35	37,295	A>C				OPG054
35	37,556	GA>AA				OPG054

Specimen No.	10-kb genomic position	SNPs	AA change	Insertion	Deletion	Gene
36	31,701	T>C	E481G			OPG047
36	33,092	A>G				OPG047
36	33,468	TC>TT				OPG048
36	37,295	A>C				OPG054
36	37,556	GA>AA				OPG054
Specimen No.	15-kb genomic position	SNPs	AA change	Insertion	Deletion	Gene
21	74,414	T>C				OPG092
21	75,121		Yes		75,121	OPG092
21	82,921	A>T				OPG104
21	83,099	C>A				OPG104
21	83,190	T>C				OPG104
21	83,326	T>G				OPG105
21	83,710	T>C				OPG105
21	84,175	T>C				OPG105
21	86,151	A>G				OPG105
21	86,773	TC>TT				OPG105
21	86,850	A>T				OPG105
22	74,414	T>C				OPG092
22	75,121		Yes		75,121	OPG092
22	77,117	TC>TT				OPG095
22	83,710	T>C				OPG105
22	86,773	TC>TT				OPG105
22	86,850	A>T	Q 1180 L			OPG105
23	74,414	T>C				OPG092
23	75,121		Yes		75,121	OPG092
23	83,710	T>C				OPG105
23	84,175	T>C				OPG105
23	86,773	TC>TT				OPG105
24	74,414	T>C				OPG092
24	75,121		Yes		75,121	OPG092
24	83,710	T>C				OPG105
24	84,175	T>C				OPG105
24	86,773	TC>TT				OPG105
25	74,414	T>C				OPG092
25	75,121		Yes		75,121	OPG092
25	83,710	T>C				OPG105
25	86,773	TC>TT				OPG105
26	74,414	T>C				OPG092
26	75,121		Yes		75,121	OPG092
26	83,710	T>C				OPG105
26	84,175	T>C				OPG105
26	86,773	TC>TT				OPG105
27	74,414	T>C				OPG092
27	75,121		Yes		75,121	OPG092
27	83,710	T>C				OPG105
27	86,773	TC>TT				OPG105
27	86,850	A>T	Q 1180 L			OPG105
28	74,414	T>C				OPG092
28	75,121		Yes		75,121	OPG092
28	77,117	TC>TT				OPG095
28	83,710	T>C				OPG105
28	86,773	TC>TT				OPG105
28	86,850	A>T	Q 1180 L			OPG105
29	74,414	T>C				OPG092
29	75,121		Yes		75,121	OPG092
29	76,366	G>C				OPG094
29	76,404	C>G				OPG094
29	82,894	TC>TT				OPG104
29	83,710	T>C				OPG105
29	86,773	TC>TT				OPG105
29	86,850	A>T	Q 1180 L			OPG105
30	74,414	T>C				OPG092
30	75,121		Yes		75,121	OPG092
30	76,217	C>A				OPG094
30	80,872	T>C				OPG101
30	83,710	T>C				OPG105
30	88,672	T>C				OPG101
30	86,773	TC>TT				OPG105
30	86,850	A>T	Q 1180 L			OPG105

Specimen No.	10-kb genomic position	SNPs	AA change	Insertion	Deletion	Gene
31	74,414	T>C				OPG092
31	75,121		lose 4A-		75,121	OPG092
31	78,168	G>A				OPG097
31	79,829	GA>AA				OPG098
31	83,710	T>C				OPG105
31	84,175	T>C				OPG105
31	86,773	TC>TT				OPG105
31	86,850	A>T	Q 1180 L			OPG105
31	88,529	G>A				OPG108
32	74,414	T>C				OPG092
32	75,121		lose 4A-		75,121	OPG092
32	78,168	G>A				OPG097
32	79,829	GA>AA				OPG098
32	83,710	T>C				OPG105
32	86,773	TC>TT				OPG105
32	86,850	A>T	Q 1180 L			OPG105
32	88,529	G>A				OPG108
33	74,414	T>C				OPG092
33	75,121		lose 4A-		75,121	OPG092
33	78,168	G>A				OPG097
33	79,829	GA>AA				OPG098
33	83,710	T>C				OPG105
33	86,773	TC>TT				OPG105
33	88,529	G>A				OPG108
34	74,414	T>C				OPG092
34	75,121		lose 4A-		75,121	OPG092
34	78,168	G>A				OPG097
34	79,829	GA>AA				OPG098
34	83,710	T>C				OPG105
34	86,773	TC>TT				OPG105
34	88,529	G>A				OPG108
35	74,414	T>C				OPG092
35	75,121		lose 4A-		75,121	OPG092
35	78,168	G>A				OPG097
35	79,829	GA>AA				OPG098
35	83,710	T>C				OPG105
35	86,773	TC>TT				OPG105
35	86,850	A>T	Q 1180 L			OPG105
35	88,529	G>A				OPG108
36	74,414	T>C				OPG092
36	75,121		lose 4A-		75,121	OPG092
36	78,168	G>A				OPG097
36	79,829	GA>AA				OPG098
36	83,710	T>C				OPG105
36	86,773	TC>TT				OPG105
36	86,850	A>T	Q 1180 L			OPG105
36	88,529	G>A				OPG108

*Specimens No.1-20 were from the USA; No.21-36 were from DRC. The 10-kb and the 15-kb genomic positions were referenced to MPXV genome ON563414.3 for the specimens from the USA and to MPXV genome KC257460 for the specimens from DRC respectively. SNPs: single nucleotide polymorphisms. AA: amino acid. OPG: orthopoxvirus gene.

Appendix Table 6. Conserved SNPs over the 10-kb and the 15-kb regions of the published MPXV genomes (n=88)*

Clade	Conserved SNP	Gene	Clade Ia (n=25)	Clade Ib (n=16)	Clade IIa (n=12)	Clade IIb lineage A (n=20)	Clade IIb Lineage B (n=15)
Clade I	G29923A	OPG047	Yes	Yes	No	No	No
	C30730T	OPG047	Yes	Yes	No	No	No
	C31081T	OPG047	Yes	Yes	No	No	No
	A31102G	OPG047	Yes	Yes	No	No	No
	C31529T	OPG048	Yes	Yes	No	No	No
	T32867C	OPG049	Yes	Yes	No	No	No
	T33102A	OPG049	Yes	Yes	No	No	No
	C33159T	noncoding region	Yes	Yes	No	No	No
	A33656C	noncoding region	Yes	Yes	No	No	No
	T36049C	OPG055	Yes	Yes	No	No	No
	G36445A	OPG055	Yes	Yes	No	No	No
	C37170T	OPG056	Yes	Yes	No	No	No
	T39248C	OPG057	Yes	Yes	No	No	No
	A73076G	OPG093	Yes	Yes	No	No	No
	C73350T	OPG093	Yes	Yes	No	No	No
	A75790G	OPG096	Yes	Yes	No	No	No
	T76123C	OPG097	Yes	Yes	No	No	No
	A76198G	OPG097	Yes	Yes	No	No	No
	A76648G	OPG097	Yes	Yes	No	No	No
	A76911G	OPG098	Yes	Yes	No	No	No
	A78461C	OPG100	Yes	Yes	No	No	No
	A78942G	OPG101	Yes	Yes	No	No	No
	C79267T	OPG102	Yes	Yes	No	No	No
	C79502A	OPG102	Yes	Yes	No	No	No
	A79829G	OPG102	Yes	Yes	No	No	No
	T82688C	OPG105	Yes	Yes	No	No	No
	T83360C	OPG105	Yes	Yes	No	No	No
	T83843C	OPG105	Yes	Yes	No	No	No
	T84290C	OPG105	Yes	Yes	No	No	No
	A84395G	OPG105	Yes	Yes	No	No	No
	C85763T	OPG107	Yes	Yes	No	No	No
	A86247G	OPG107	Yes	Yes	No	No	No
	G86502T	OPG108	Yes	Yes	No	No	No
	G86728A	OPG108	Yes	Yes	No	No	No
Clade Ia	C35352A	OPG054	Yes	No	No	No	No
Clade Ib	G75982A	OPG097	No	Yes	No	No	No
	G77643A	OPG098	No	Yes	No	No	No
	G86343A	OPG108	No	Yes	No	No	No
Clade IIa	C30510T	OPG047	No	No	Yes	No	No
	G30699T	OPG047	No	No	Yes	No	No
	C35859T	OPG054	No	No	Yes	No	No
	C36732T	OPG055	No	No	Yes	No	No
	T38382A	OPG056	No	No	Yes	No	No
Clade IIb	G30367A	OPG047	No	No	No	No	Yes
Lineage B	G31053A	OPG047	No	No	No	No	Yes
	G34459A	OPG053	No	No	No	No	Yes
	G37202A	OPG056	No	No	No	No	Yes
	C38662T	OPG056	No	No	No	No	Yes
	C39139T	OPG057	No	No	No	No	Yes

*Twenty-five MPXV genomes belonged to clade Ia; 16 genomes belonged to clade Ib; 12 genomes belonged to IIa; 15 genomes belonged to clade IIb lineage B, and 20 genomes belonged to clade IIb lineage A. SNP: single nucleotide polymorphisms. OPG: orthopoxvirus gene.

Appendix Table 7. Subclades and lineages of MPXV in clinical specimens (n=49) from the USA and DRC, assigned by Nextclade*

Specimen Number	Subclade	Lineage	Verified by WGS
1	IIb	B.1	Yes
2	IIb	B.1	Yes
3	IIb	B.1.20	Yes
4	IIb	B.1	Yes
5	IIb	B.1	Yes
6	IIb	B.1	Yes
7	IIb	B.1	Yes
8	IIb	B.1	Yes
9	IIb	B.1.20	Yes
10	IIb	B.1	Yes
11	IIb	B.1.20	Yes
12	IIb	B.1	Yes
13	IIb	B.1	Yes
14	IIb	B.1	Yes
15	IIb	B.1.20	Yes
16	IIb	B.1.20	Yes
17	IIb	B.1.20	Yes
18	IIb	B.1.20	Yes
19	IIb	B.1	Not performed
20	IIb	B.1	Not performed
21	Ia	N/A	Yes
22	Ia	N/A	Yes
23	Ia	N/A	Not performed
24	Ia	N/A	Yes
25	Ia	N/A	Yes
26	Ia	N/A	Yes
27	Ia	N/A	Yes
28	Ia	N/A	Not performed
29	Ia	N/A	Yes
30	Ia	N/A	Yes
31	Ib	N/A	Yes
32	Ib	N/A	Yes
33	Ib	N/A	Yes
34	Ib	N/A	Yes
35	Ib	N/A	Yes
36	Ib	N/A	Yes
37	IIb	B.1	Yes
38	IIb	B.1	Not performed
39	IIb	B.1	Yes
40	IIb	B.1	Yes
41	IIb	B.1	Yes
42	IIb	B.1.7	Yes
43	IIb	B.1	Yes
44	IIb	B.1	Yes
45	IIb	B.1.3	Yes
46	IIb	B.1	Not performed
47	IIb	B.1	Not performed
48	IIb	B.1	Not performed
49	IIb	B.1	Failed

*WGS: whole genome sequencing. N/A: not available.

Appendix Table 8. The 10-kb and the 15-kb region-based subclade and lineage assignment of 88 published MPXV genomes using the NextFlow pipeline, with GenBank accession number per genome. Clade Ia had 25 genomes, clade Ib had 16 genomes, clade IIa had 12 genomes, clade IIb lineage B had 15 genomes, and clade IIb lineage A had 20 genomes.

GenBank accession	Subclade	GenBank accession	Subclade / lineage
DQ011155.1	Ia	DQ011153.1	IIa
JX878407.1	Ia	KJ136820.1	IIa
JX878409.1	Ia	MN346702	IIa
JX878410.1	Ia	MN346696	IIa
JX878417.1	Ia	KJ642616.1	IIa
JX878418.1	Ia	AY603973.1	IIa
KJ642618.1	Ia	AY741551.1	IIa
MN702444.1	Ia	DQ011156.1	IIa
MN702445.1	Ia	MN346698.1	IIa
NC_003310.1	Ia	MN346690.1	IIa
PP601190.1	Ia	MT903348	IIa
PQ221532.1	Ia	MN346691.1	IIa
PQ221557.1	Ia	LC753969.1	IIb.C.1
PQ221562.1	Ia	ON880413.1	IIb.B.1.12
PQ221580.1	Ia	ON927248.1	IIb.B.1.7
PQ221583.1	Ia	ON959143.1	IIb.B.1.10
PQ221595.1	Ia	OP160532.1	IIb.B.1
PQ221607.1	Ia	OR464191	IIb.B.1.2
PQ221610.1	Ia	OR500078.1	IIb.B.1.1
PQ221666.1	Ia	OR759176.1	IIb.B.1.4
PQ221727.1	Ia	OQ331000	IIb.B.1.17
PQ221749.1	Ia	OX009124.1	IIb.B.1.14
PQ221858.1	Ia	PP265943.1	IIb.B.1
PQ352025.1	Ia	PP481199.1	IIb.C.1.1
PQ352027.1	Ia	PP648208.1	IIb.C.1.1
PP601208.1	Ib	PQ152020	IIb.B.1.20
PP601209.1	Ib	PQ153127.1	IIb.B.1.20
PP601214.1	Ib	ON676708.1	IIb.A.1.1
PP601228.1	Ib	OP413718.1	IIb.A.3
PQ178862.1	Ib	OP535313.1	IIb.A
PQ220056.1	Ib	OP535318.1	IIb.A
PQ220060.1	Ib	OP535321.1	IIb.A
PQ221884.1	Ib	OP535333.1	IIb.A
PQ221893.1	Ib	OP612677.1	IIb.A.3
PQ221895.1	Ib	OP612682.1	IIb.A.1
PQ221912.1	Ib	OP612686.1	IIb.A.1
PQ240043.1	Ib	OP612690.1	IIb.A.2
PQ305786.1	Ib	OP612691.1	IIb.A.2
PQ305791.1	Ib	PP852955.1	IIb.A.2.2
PQ305798.1	Ib	PP852959.1	IIb.A.2.3
PQ305800.1	Ib	PP852992.1	IIb.A.2.3
		PP853001.1	IIb.A.2.3
		PP853026.1	IIb.A.2.1
		PP853039.2	IIb.A.2
		PP853053.1	IIb.A.2
		PP860028.1	IIb.A
		KJ642617.1	IIb.A

Appendix Table 9. The 10-kb and the 15-kb amplicon-based assignment of MPXV in the clinical specimens from the USA and DRC (n=49) verified using the NextFlow pipeline.

Specimen No.	Subclade / lineage
1	IIb, B.1
2	IIb, B.1
3	IIb, B.1.20
4	IIb, B.1
5	IIb, B.1
6	IIb, B.1
7	IIb, B.1
8	IIb, B.1.20
9	IIb, F.4
10	IIb, E.1
11	IIb, F.4
12	IIb, E.1
13	IIb, B.1.9
14	IIb, F.4
15	IIb, F.4
16	IIb, F.4
17	IIb, F.4
18	IIb, F.4
19	IIb, F.1
20	IIb, F.1
21	Ia
22	Ia
23	Ia
24	Ia
25	Ia
26	Ia
27	Ia
28	Ia
29	Ia
30	Ia
31	Ib
32	Ib
33	Ib
34	Ib
35	Ib
36	Ib
37	IIb, B.1
38	IIb, B.1
39	IIb, B.1
40	IIb, B.1
41	IIb, B.1
42	IIb, B.1
43	IIb, B.1
44	IIb, B.1
45	IIb, B.1.3
46	IIb, B.1
47	IIb, B.1
48	IIb, B.1
49	IIb. B.1

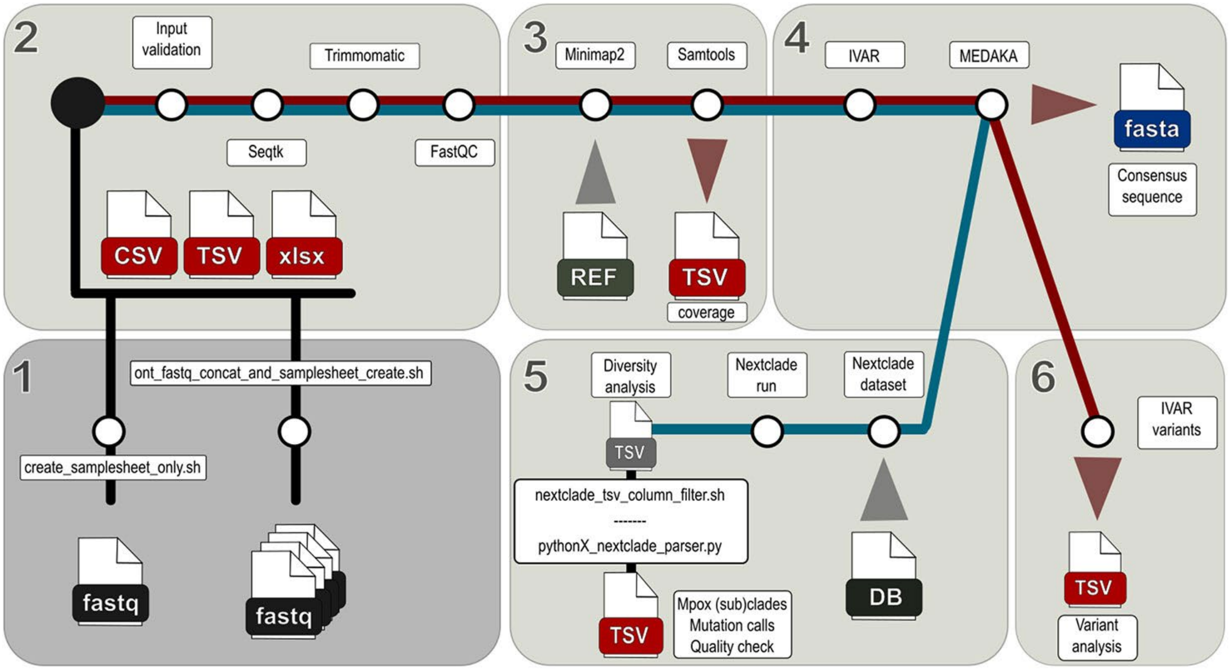

Appendix Figure 1. Diagram of the Nextflow pipeline.1. Samplesheet creation; 2. Pre-processing and QC; 3. Reference-based assembly and refinement; 4. Consensus generation and polishing; 5. Nextclade run; 6. Variant table analysis.

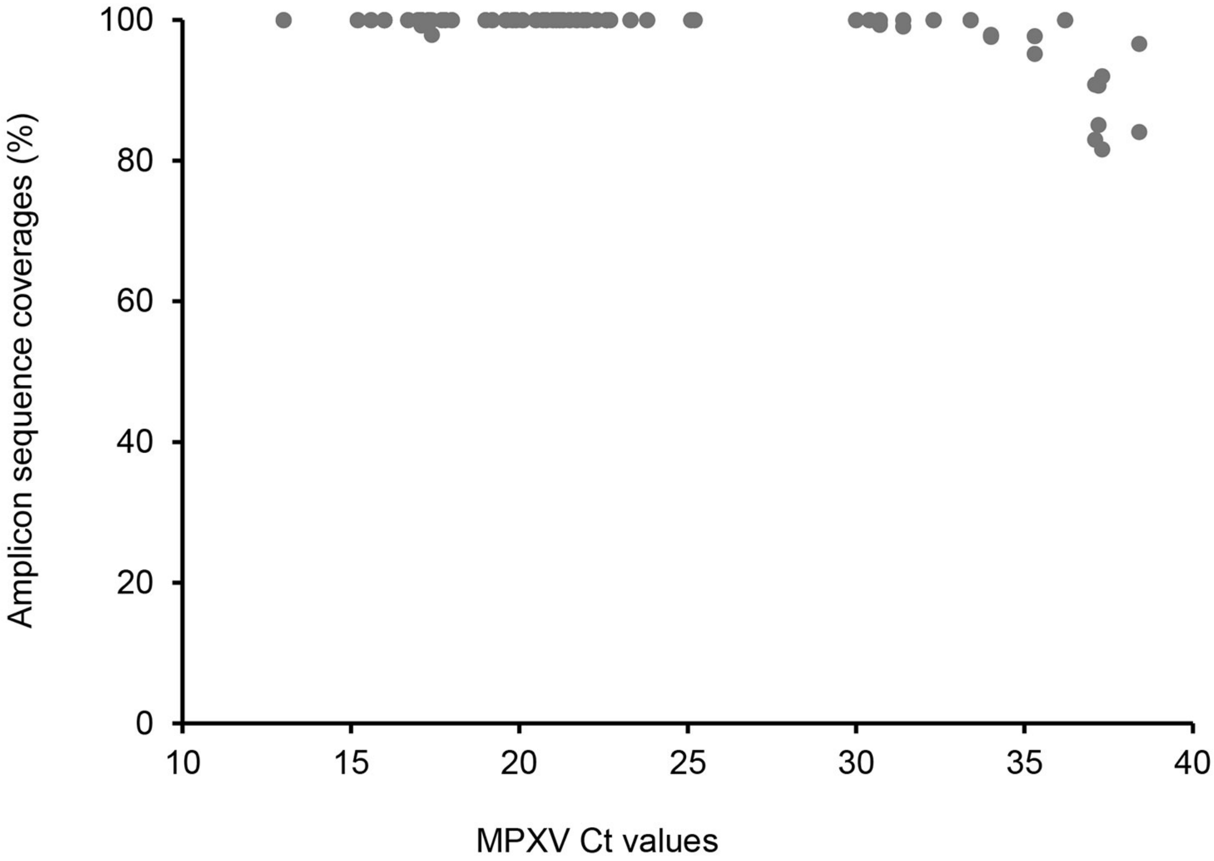

Appendix Figure 2. Correlation of MPXV Ct values with the 10-kb and the 15-kb region sequence coverages. Ct values ranged from 13.2 to 38.4. Amplicon sequence coverages were between 68.6% and 100% over the 10-kb or the 15-kb region. MPXV Ct values below 35 provided high sequence coverages above 98% over the two regions.
